# Heptameric Targeting Ligands against EGFR and HER2 with High Stability and Avidity

**DOI:** 10.1371/journal.pone.0043077

**Published:** 2012-08-09

**Authors:** Dongwook Kim, Yitang Yan, C. Alexander Valencia, Rihe Liu

**Affiliations:** 1 Division of Chemical Biology and Medicinal Chemistry, UNC Eshelman School of Pharmacy, University of North Carolina, Chapel Hill, North Carolina, United States of America; 2 Carolina Center for Genome Sciences, University of North Carolina, Chapel Hill, North Carolina, United States of America; Aligarh Muslim University, India

## Abstract

Multivalency of targeting ligands provides significantly increased binding strength towards their molecular targets. Here, we report the development of a novel heptameric targeting system, with general applications, constructed by fusing a target-binding domain with the heptamerization domain of the Archaeal RNA binding protein Sm1 through a flexible hinge peptide. The previously reported affibody molecules against EGFR and HER2, Z^EGFR^ and Z^HER2^, were used as target binding moieties. The fusion molecules were highly expressed in *E. coli* as soluble proteins and efficiently self-assembled into multimeric targeting ligands with the heptamer as the predominant form. We demonstrated that the heptameric molecules were resistant to protease-mediated digestion or heat- and SDS-induced denaturation. Surface plasmon resonance (SPR) analysis showed that both heptameric Z^EGFR^ and Z^HER2^ ligands have a significantly enhanced binding strength to their target receptors with a nearly 100 to 1000 fold increase relative to the monomeric ligands. Cellular binding assays showed that heptameric ligands maintained their target-binding specificities similar to the monomeric forms towards their respective receptor. The non-toxic property of each heptameric ligand was demonstrated by the cell proliferation assay. In general,, the heptamerization strategy we describe here could be applied to the facile and efficient engineering of other protein domain- or short peptide-based affinity molecules to acquire significantly improved target-binding strengths with potential applications in the targeted delivery of various imaging or therapeutic agents..

## Introduction

Target binding affinity molecules are of great importance in various biomedical applications. One of the major challenges in developing targeting ligands is to improve their target-binding strength while maintaining their high specificity. Although such properties can be improved through extensive affinity maturation, the process is slow, tedious, and often limited [1]. Currently, there is an urgent need for the facile development of affinity molecules that can bind to the targets of interest with high affinity and specificity. One of the most critical design parameters for satisfactory *in vivo* targeting is to increase targeting ligand valency, defined as the number of antigen-binding sites [1]. Multivalent targeting for the attainment of high binding affinity has known natural examples, such as the binding between an antibody and its target antigen: an intrinsic characteristic of mammalian antibodies [1]. Multivalent targeting ligands maintain several major advantages over monovalent ligands when targeting cell surface receptors. First, the target-binding strength of the multivalent ligand could be significantly improved [2]. Second, the multimerization process increases the molecular weight of the affinity molecule above that of the glomerular filtration cut-off, thereby reducing *in vivo* excretion while increasing tumor accumulation characteristics via enhanced permeability and retention (EPR). For example, it has demonstrated that monovalent binding is often not sufficient for efficacious cancer targeting, and most monovalent targeting ligands, despite nanomolar or picomolar binding affinities, tend to have fast dissociation rates, providing modest retention times on the target antigen in *in vivo* non-equilibrium environments [1]–[3].

Due to these advantages, several techniques in multivalency engineering of antibodies have been developed, including domain-swapping, linear fusion, chemical linking, self-assembly, and heterodimerization [1]. Most of these approaches are limited to targeting ligands based on natural antibodies or their fragments. However, a self-assembly approach based on the use of various domains that permit self-multimerization may be a general strategy for the systematic development of novel targeting ligands. Recently, the self-assembly strategy has been successfully applied, by exploring several multimerization domains, in generating multivalent antibody fragments. These multimerization domains includes TNF-alpha for the formation of homotrimers, the amphipathic helix of GCN4, the multimerization peptide of p53 and the core domain of streptavidin for the formation of tetramers, and the coiled-coil assembly domain of cartilage oligomeric matrix protein (COMP) and the B-subunit of bacterial verotoxin for the formation of pentamers [4]–[11].

Successful and efficient conversion of a monovalent ligand into a multivalent form is challenging and requires a combination of unique features on both the target binding and the multimerization moieties. Due to the tendency of aggregation, steric hindrance, and fast dissociation, only few self-multimerization domains are suitable for efficient self-assembly [1]. First, the scaffold should be small and soluble enough for high expression in bacteria. Second, the self-assembly of a monomeric domain into its multimeric form with desired valency should be very efficient with extraordinarily high association constants and low aggregation tendency. The resulting complex should have a well-defined parallel multimeric structure with high stability that allows for the introduction of target-binding moiety and hinge region to achieve desired multivalency without disrupting target binding. This is particularly challenging when the complex is significantly diluted in the bloodstream under *in vivo* conditions. To circumvent these problems, new multimerization domains require investigation for the development of higher avidity targeting ligands.??

**Figure 1:**
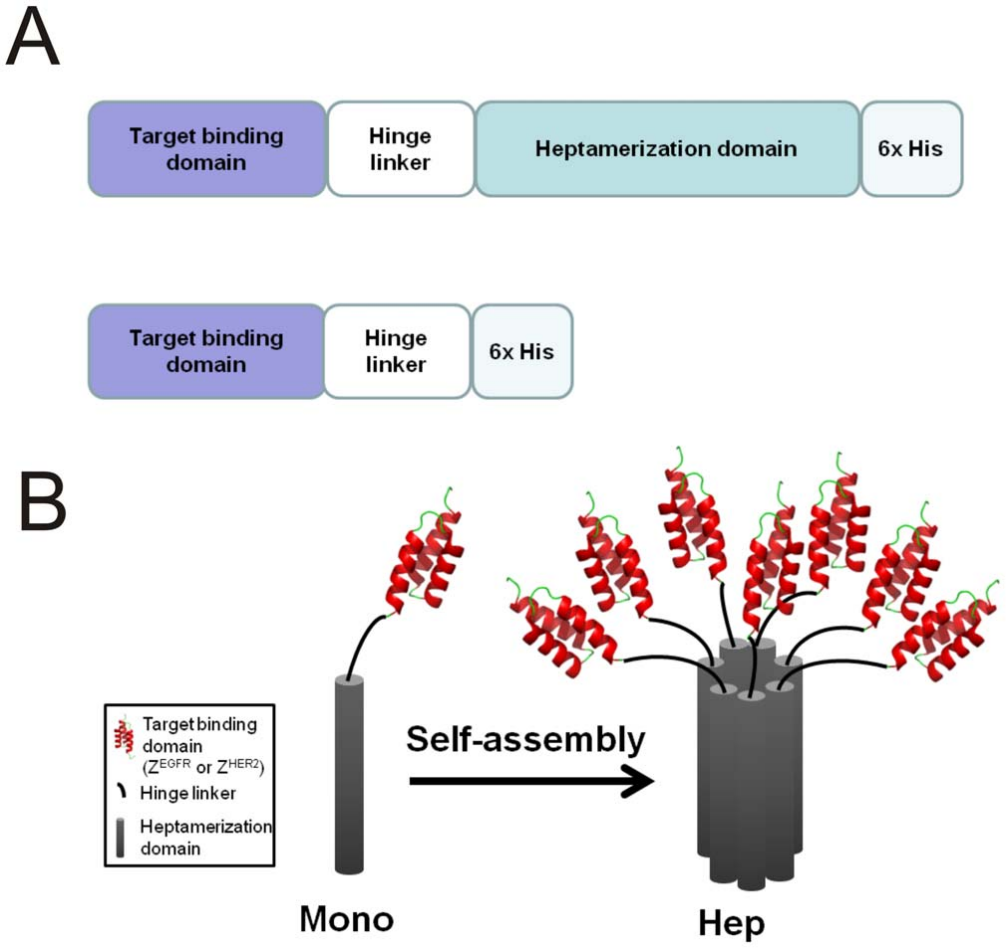
Schematic diagram of monomeric and heptameric targeting ligands. (A) The cDNA of heptameric ligand consists of coding regions for a target binding domain, a flexible hinge linker, and a heptamerization domain. A 6×His-tag was introduced on the C-terminus of each molecule. The structure of the monomeric targeting ligand is similar to the heptameric ligand with the exception of the absence of the heptamerization domain. (B) Schematic representation of the monomeric and heptameric ligands;; Affibody (Z domain) structure was obtained from PDB database (PDB ID:2B89).

**Table 1:** Amino acid sequences of each component of the heptameric targeting ligands.

	Amino acids
**Z^EGFR^**	MVDNKFNKEM WAAWEEIRNL PNLNGWQMTAFIASLVDDPS QSANLLAEAK KLNDAQAPK
**Z^HER2^**	MVDNKFNKEM RNAYWEIALL PNLNNQQKRAFIRSLYGDPS QSANLLAEAK KLNDAQAPK
**Hinge Linker**	GPQPQPKPQPK PEPEPQPQGG
**Heptamerization domain**	MPPRPLDVLN RSLKSPVIVR LKGGREFRGT LDGYDIHMNL VLLDAEEIQN GEVVRKVGSVVIRGDTVVFV SPAPGGE

**Figure 2:**
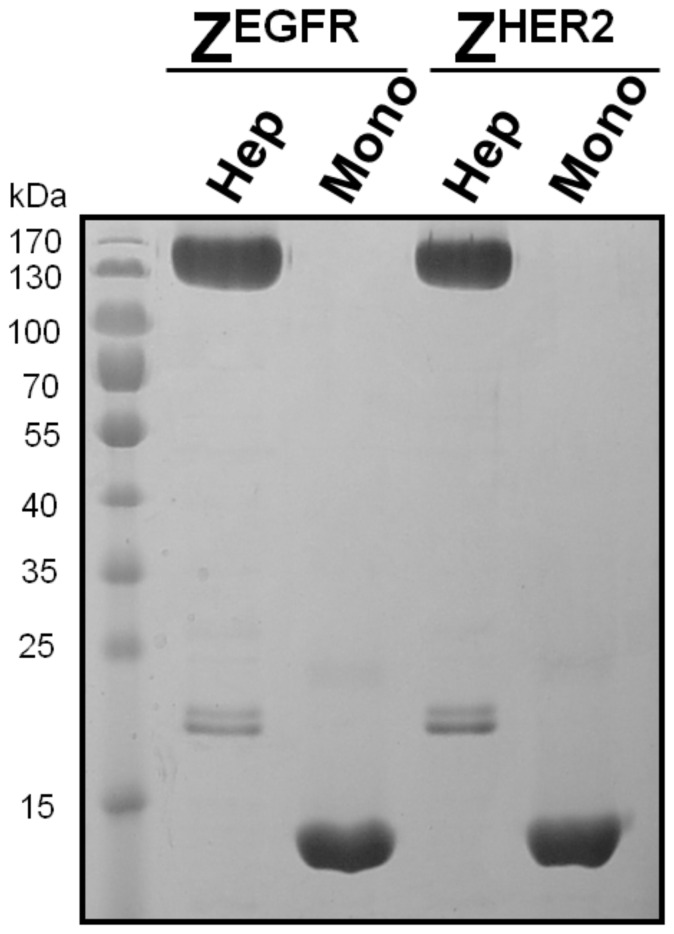
SDS-PAGE analysis of the purified monomeric and heptameric targeting ligands. The purified heptameric Z^EGFR^, monomeric Z^EGFR^, heptameric Z^HER2^, and monomeric Z^HER2^ligands were separated on a 10% SDS-PAGE gel. About 5 µg of each protein was applied to each lane.

**Figure 3:**
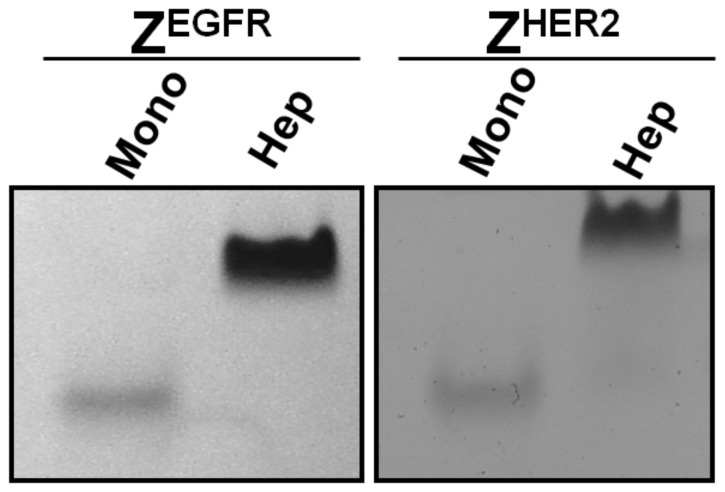
Native gel separation of monomeric and heptameric targeting ligands. The purified monomeric Z^EGFR^, heptameric Z^EGFR^, monomeric Z^HER2^ and heptameric Z^HER2^ ligands were separated on an 8% native gel. About 5 µg of the purified monomer or 20 µg heptamer was loaded to the appropriate lane.

**Figure 4:**
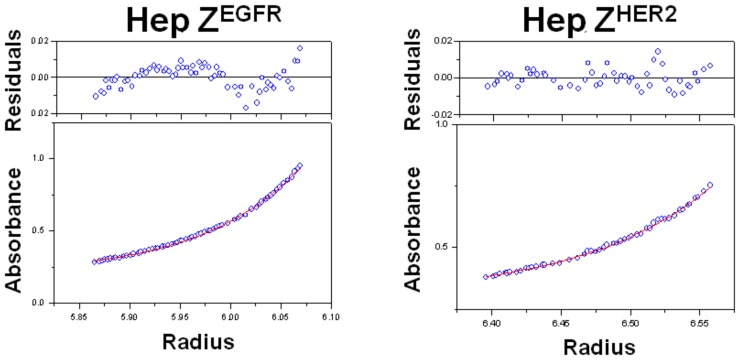
Determination of the molecular weights of the heptameric targeting ligands by analytical ultracentrifugation analysis. Purified heptameric Z^EGFR^ and heptameric Z^HER2^ ligands were centrifuged at 10,000 g for 20 h. Absorbances at 280 nm were recorded every two hours.

**Figure 5:**
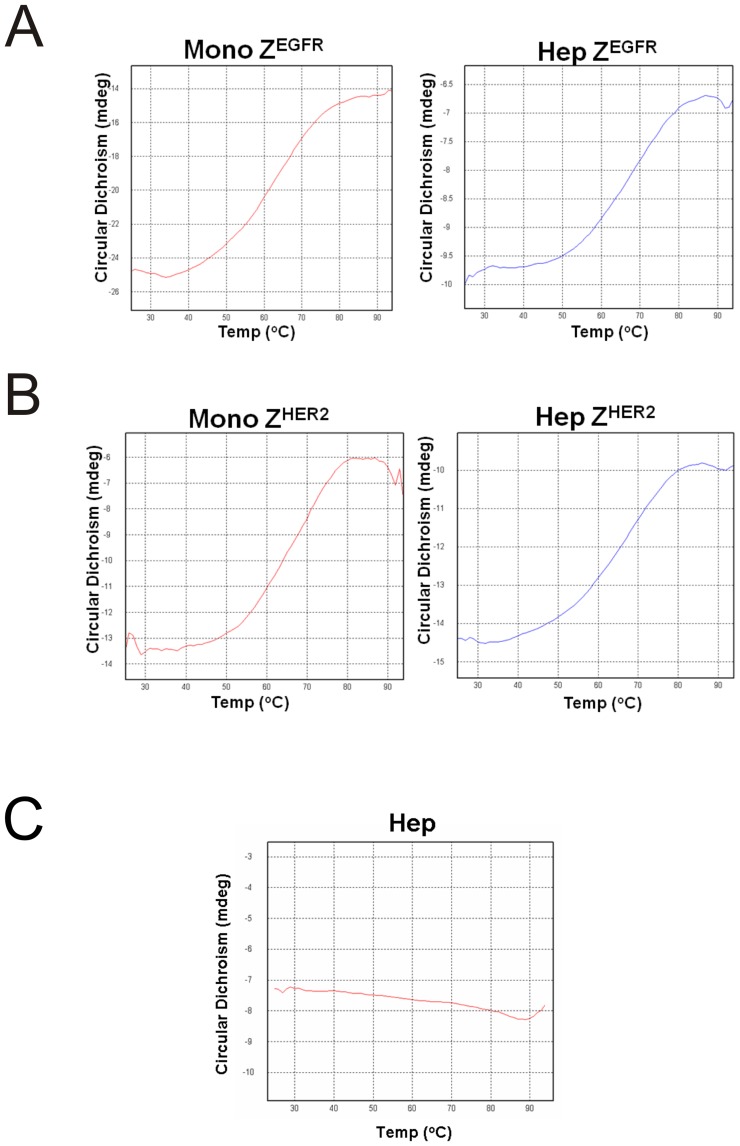
Heat stability assessment of the monomer and the heptamer by circular dichroism analysis. (A) Monomeric and heptameric Z^EGFR^, (B) monomeric and heptameric Z^HER2^ targeting ligands, and (C) heptameric core itself were prepared in a 10 mM phosphate buffer, pH 7.4. Temperature was increased from 25°C to 94°C. Spectra were recorded at various temperatures. The ellipticity at 220 nm was used for the analysis.

**Figure 6:**
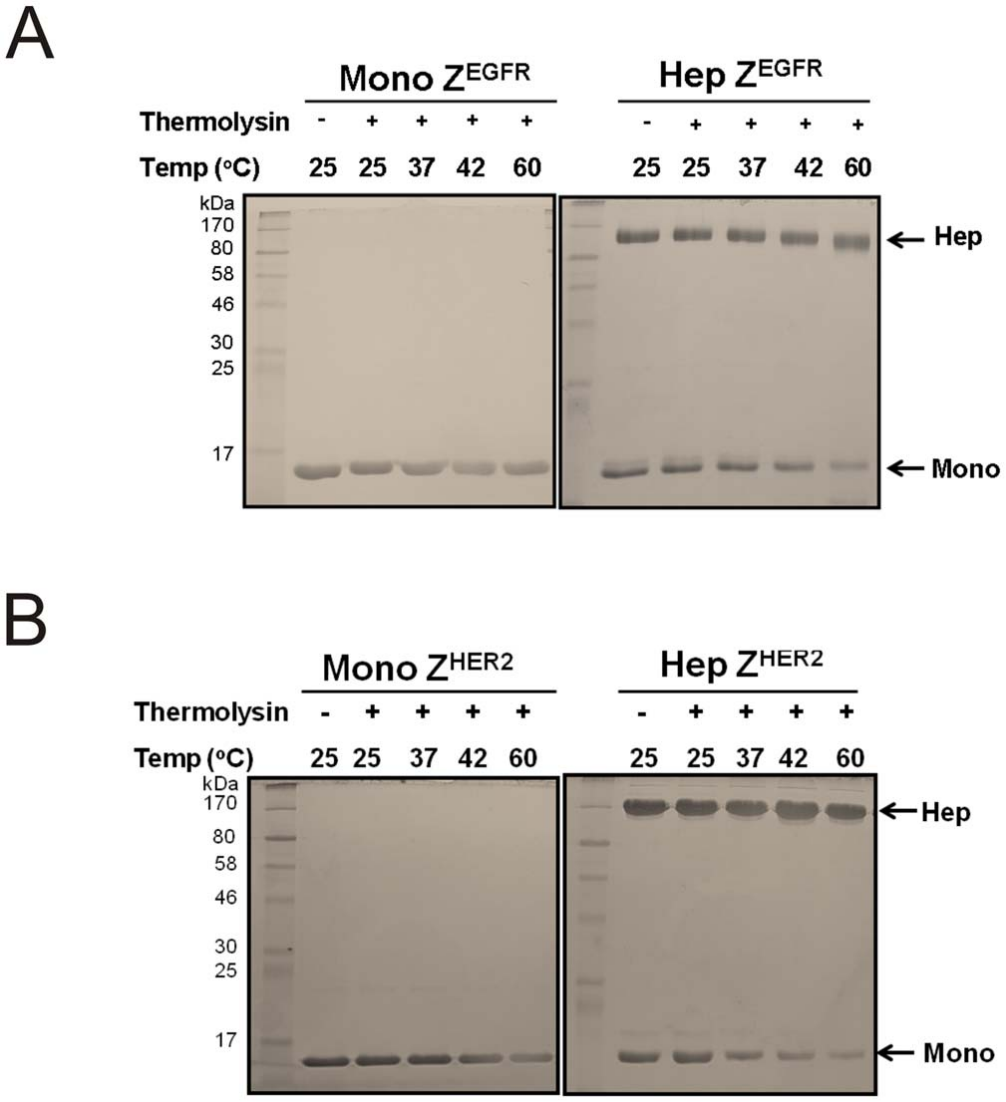
Analysis of the protease resistance of the monomer and the heptamer by thermolysin. (A) About 5 µg of monomeric and heptameric Z^EGFR^ and (B) monomeric and heptameric Z^HER2^ targeting ligands were incubated with 100 ng of thermolysin at different temperatures for 20 min. After incubation, reaction was stopped by adding SDS sample buffer and each reaction mixture was separated on a 10% SDS-PAGE to examine protein degradation.

**Figure 7:**
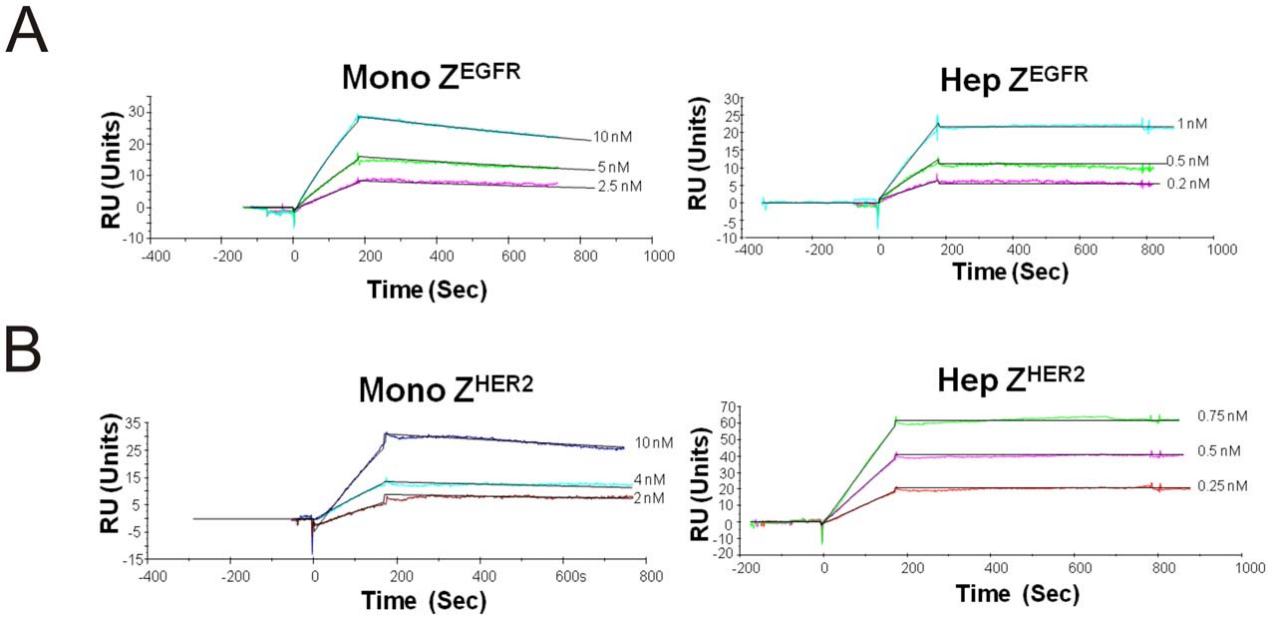
Binding dynamics of monomeric and heptameric targeting ligands by BIAcore analysis. The extracellular domain of (A) EGFR and (B) HER2 receptors were immobilized on the CM5 chip. Different concentrations of monomer or heptamer proteins were injected into the channels. Analyses were performed at room temperature at a flow rate of 20 µl/min.

**Table 2:** Binding constants of each monomeric and heptameric targeting ligand using BIAcore analysis.

	K_a_ (1/Ms)	K_d_ (1/s)	K_D_ (M)
**Z^EGFR^ monomer**	6.07×10^4^	1.59×10^−4^	2.62×10^−9^
**Z^EGFR^ heptamer**	1.91×10^4^	5.67×10^−7^	2.9×10^−11^
**Z^HER2^ monomer**	1.69×10^5^	2.95×10^−4^	1.75×10^−9^
**Z^HER2^ heptamer**	3.15×10^4^	7.15×10^−8^	2.27×10^−12^

**Figure 8:**
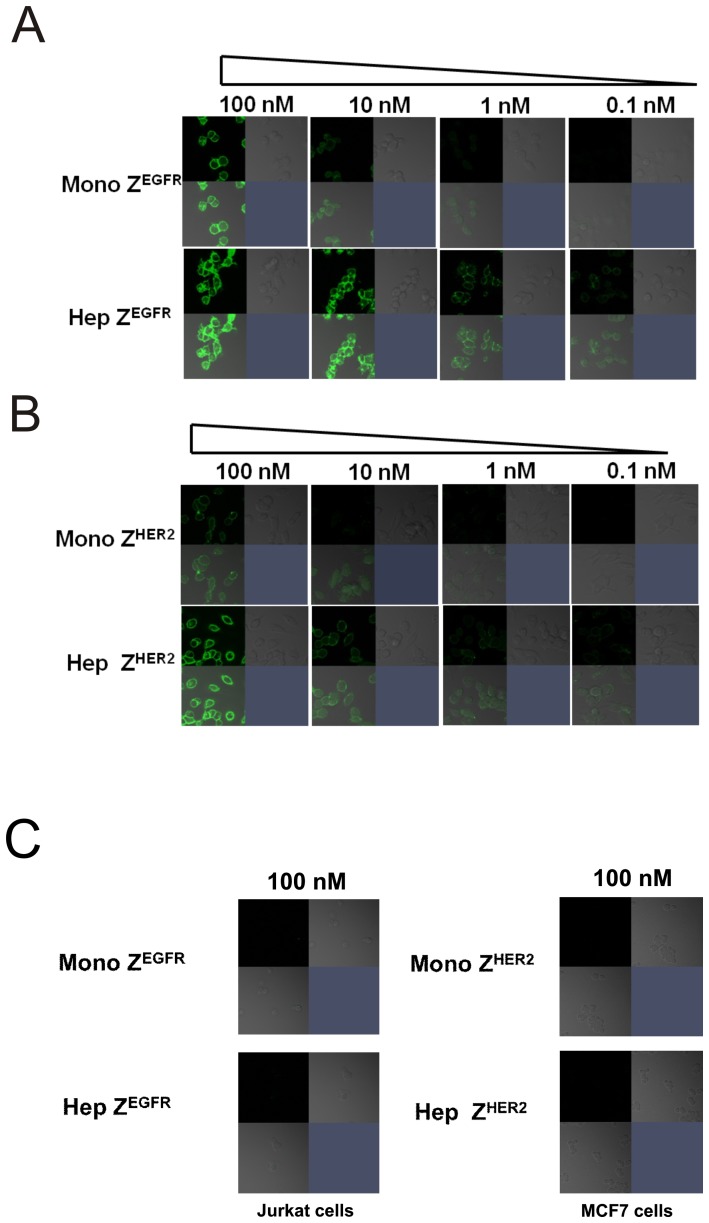
Cell-based surface receptor binding properties of the monomer and heptamer. (A) EGFR-positive A431 cells were grown on coverslips. Different concentration of FITC-labeled monomeric and heptameric Z^EGFR^ ligands was incubated with A431 cells for 30 min at 25°C. (B) HER2-positive SK-OV3 cells were grown on coverslips. FITC-labeled monomeric and heptameric Z^HER2^ ligands were incubated with SK-OV3 cells for 30 min at 25°C. (C) EGFR-negative Jurkat cells and HER2-low expressing MCF7 cells were grown on coverslips. 100 nM of FITC-labeled monomeric and heptameric ligands were incubated with Jurkat and MCF cells for 30 min at 25°C.

**Figure 9:**
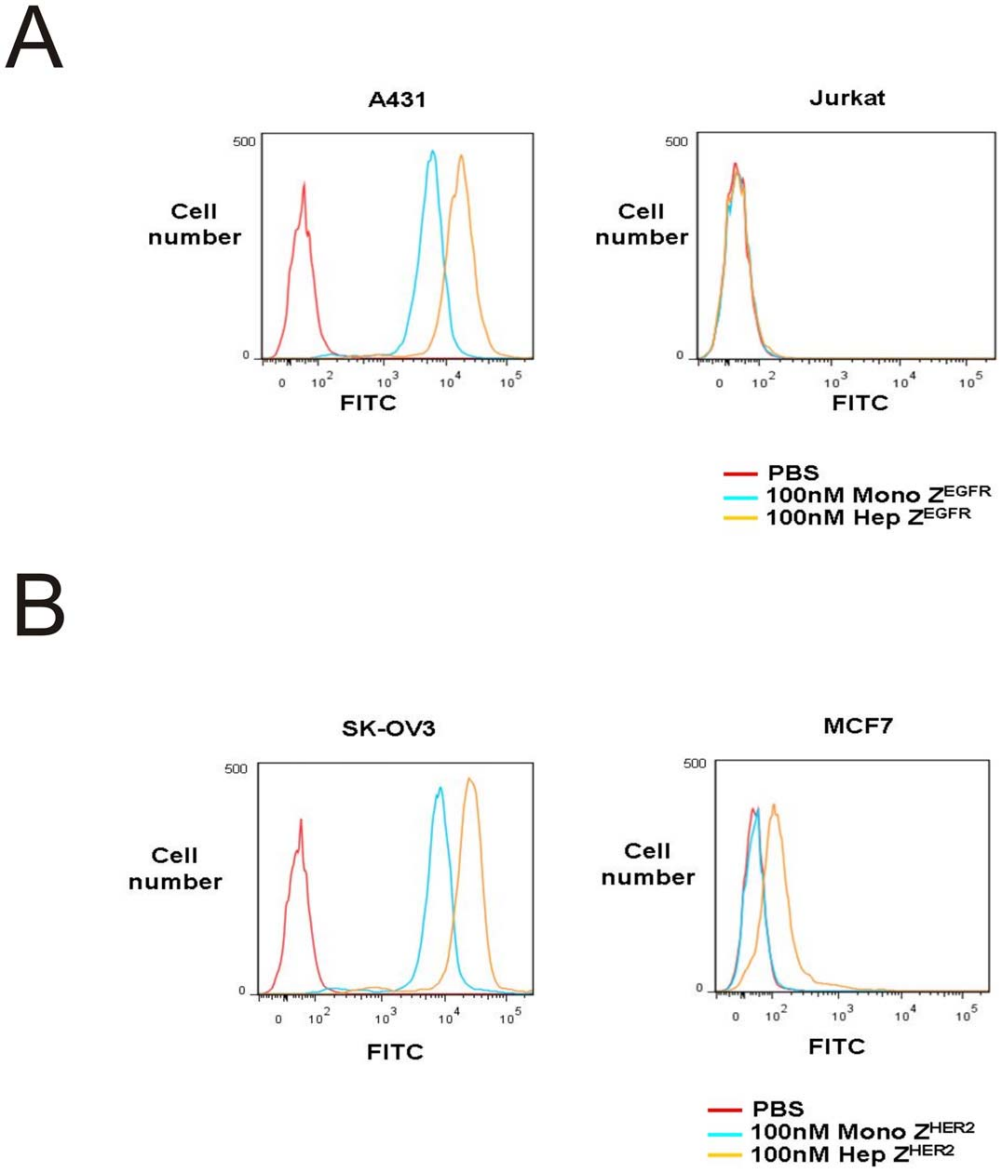
Cell binding analysis by flow cytometry. (A) 100 nM FITC-monomeric and heptameric Z^EGFR^ ligands were used for labeling of EGFR positive A431 and negative Jurkat cells, and analyzed by flow cytometry. Cells incubated with PBS were served as negative control. (B) 100 nM FITC-monomeric and heptameric Z^HER2^ ligands were used for labeling of HER2 positive SK-OV3 and HER2 low expressing MCF7 cells, and analyzed by flow cytometry. Cells incubated with PBS were served as negative control.

**Figure 10:**
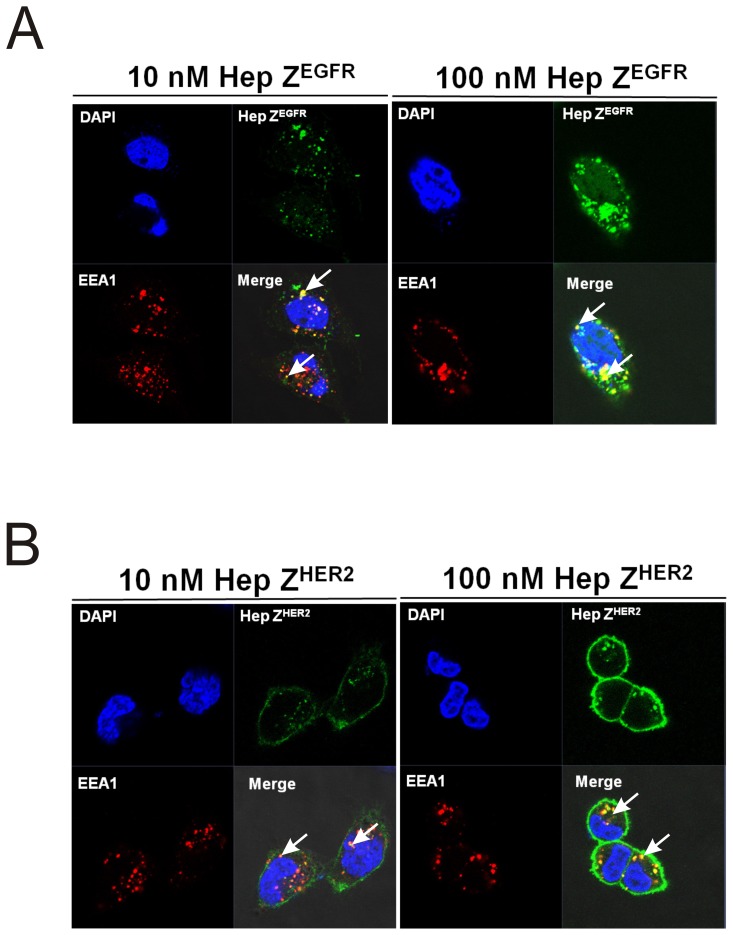
Co-localization of EEA1 and heptameric targeting ligands. (A) Two different concentrations of the FITC-labeled heptameric Z^EGFR^ targeting ligands were incubated with A431 cells for 2 h at 37°C. (B) FITC labeled heptameric Z^HER2^ targeting ligands at two concentrations were incubated with SK-OV3 cells for 2 h at 37°C. EEA1 proteins were detected by Alexa 555-conjugated secondary antibody. Top left panels: cell nuclei stained with DAPI (blue); Top right panels: FITC labeled heptamer (green); bottom left panels: EEA1 antibody (red); bottom right panels: merged image of the three stainings.

**Figure 11:**
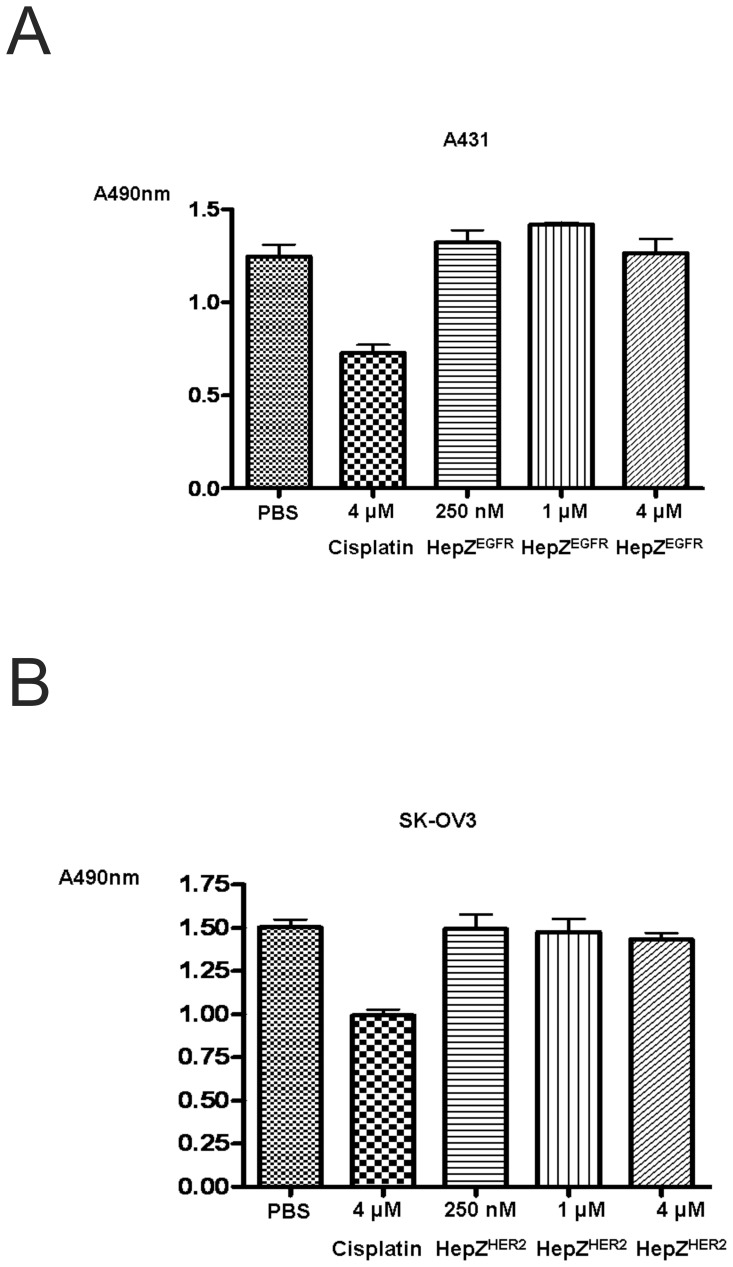
Analysis of cellular toxicity of heptameric targeting ligands by the MTS assay. A431 and SK-OV3 cells were incubated with various concentrations from 250 nM to 4 µM of each heptameric targeting ligand for 24 h at 37°C. Cisplatin (4 µM) was used as a positive control. Cells without the targeting ligands or without cisplatin were used as a negative control. Absorbance at 490 nm (A490) was measured.

Targeting ligands with di-, tri-, tetra-, and pentavalency have been developed, however, those with a valency higher than five have not been reported and it is the focus of this study [4]–[14]. One protein class featuring heptameric structures is the Sm or Sm-like (Lsm) RNA-binding protein that has been implicated in a variety of RNA processing events in all eukaryotic organisms [15]. Structural analyses indicate that the core domain of Sm protein self-assembles into a heptameric complex with a doughnut-shaped ring structure that accommodates uracil rich RNAs, as shown in human canonical Sm core domain, human Lsm, and other Sm proteins [15]. To generate novel heptameric targeting ligands with high stability and binding affinity, we chose to use the 70-amino acid multimerization domain from the hyperthermophilic Archaeal Sm protein. Recently, the crystal structures of the Sm1 and Sm2 proteins from hyperthermophilic *Archaeoglobus fulgidus* (AF) have been solved [16]. While the AF-Sm2 hexamer is RNA-dependent and only stable at low pH, the AF-Sm1 heptamer is highly stable regardless of pH and the absence of RNA. Significantly, AF-Sm1 forms a seven-membered ring, presumably due to the continuous inter-subunit hydrogen bonding between β-strands 4 and 5. The thickness of the core ring is 32 Å, while the outer and inner diameters are 65 Å and 13 Å, respectively [16].

In the present study, we have designed heptameric targeting ligands towards EGFR or HER2 receptor by utilizing the heptamerization domain of the AF-Sm1domain and EGFR- or HER2-binding Z domain as the target binding moiety connected through a flexible hinge peptide. The heptameric targeting ligands were self-assembled with high efficiency, retained their binding specificity, showed significantly enhanced target-binding strength, and demonstrated unusually high stability with non-toxic property, implying that this general heptamerization strategy has the potential to be widely applied for the systematic improvement of the target-binding strength of many affinity molecules, particularly those based on small protein domains or short peptides.

## Materials and Methods

### Cell culture

EGFR-positive A431, EGFR-negative Jurkat, HER2-positive SK-OV3, and HER2-low expressing MCF7 cells were obtained from the UNC Tissue Culture Facility. All cell lines were maintained by serial passage at 37°C in 5% CO_2_. A431 cells were grown in DMEM, Jurkat cells in RPMI1640, SK-OV3 cells in McCoy 5a, and MCF7 cells in MEM alpha media, respectively. All media were supplemented with 10% fetal bovine serum, 100 units/mL of penicillin, and 0.1 mg/mL streptomycin.

### Plasmid construction

The codon-optimized DNA sequences that code for the heptamerization domain of the Archaeal *SM1* gene, EGFR (Z^EGFR1907^), and HER2 (Z^HER2–342^) targeting affibodies were synthesized by GenScript (Piscataway, NJ). The design of each monomeric and heptameric targeting ligand is shown in Figure 1. The amino acid sequence for each component of the heptameric targeting ligands is listed in Table 1. After PCR amplification, the gene products containing the target binding domain, hinge linker, and heptamerization domain, were digested with Nco I and Xho I. The digested fragments were cloned into the corresponding sites (Nco I and Xho I) of pET28b (Novagen, Darmstadt, Germany). The cloned plasmids were confirmed by sequencing at UNC sequencing facility prior to use in protein expression.

### Protein expression and purification

Each expression vector was transformed into *E. coli* BL21 (DE3) Rosseta cells (Novagen, Darmstadt, Germany). The positive clones were selected on LB plates containing kanamycin (50 µg/mL) and chloramphenicol (34 µg/mL). A single colony was selected and grown in 5 mL of LB media overnight at 37°C. The resulting culture was added to a flask with 500 mL of LB media containing kanamycin (50 µg/mL) and chloramphenicol (34 µg/mL). The cells were grown at 37°C until the optical density (at 600 nm) reached 0.5 to 1.0. IPTG with a final concentration of 1 mM was then added to the cell cultures, followed by incubation at 22°C for 16 h. After induction, the cells were spun down at 3,000 g for 10 min at 4°C, and the pellet was stored at −20°C until use. To purify the monomeric and heptameric ligands, the cell pellet was resuspended in buffer A (25 mM HEPES pH 7.4 and 300 mM NaCl) and sonicated for 1 min for a total of 5 times. The soluble fraction was recovered by centrifugation at 12,000 g for 10 min at 4°C. The resulting fraction was loaded onto a TALON metal affinity column (Clontech, Mountainview, CA) pre-equilibrated with buffer A. Approximately 20 column volumes of buffer A were used for initial washing followed by extensive washing (20 column volumes) with buffer B (buffer A with 20 mM imidazole). The protein of interest was eluted with buffer C (buffer A with 200 mM imidazole). The quality of the purified proteins was examined by SDS-PAGE.

### Native gel electrophoresis

An 8% discontinuous native gel was prepared without SDS and reducing agents based on the standard Laemmli SDS-PAGE protocol. About 5 µg of highly purified monomer or 20 µg heptamer was loaded to the appropriate lane, and separated on an 8 % native gel. Proteins were stained with coomassie brilliant blue R-250.

### Analytical ultracentrifugation

Highly purified monomeric or heptameric ligands were prepared in a buffer containing 25 mM HEPES pH 7.4 and 150 mM NaCl. The solution was centrifuged at 10,000 g for 20 h at 20°C. The absorbance at 280 nm was recorded every 2 h during centrifugation. Each resulting absorbance was fit into a self-association model to calculate the molecular weight.

### FlTC labeling of monomeric and heptameric targeting ligands

Each monomeric and heptameric molecule was labeled with fluorescein isothiocyanate (FITC) (ACROS organics, Geels, Belgium) in 50 mM borate buffer (pH 8.5). Briefly, 1 mg of each protein was reacted with a 25 molar excess of FITC in the reaction buffer and incubated at room temperature for 2 h. The resulting mixture was quenched by the addition of 100 mM Tris-HCl (pH 8.8) at room temperature for 1 h. Un-reacted free FITC molecules were removed by passing the reaction mixture through a NAP-10 column (GE Healthcare, Piscataway, NJ). Extensive dialysis was performed overnight using a 3 kDa molecular weight cut off dialysis membrane (GE Healthcare, Piscataway, NJ) to further remove the residual FITC.

### Cell surface binding analysis

Approximately 2×10^4^ cells were seeded on coverslips and allowed to grow in the appropriate media for 16 h. The resulting coverslips were washed twice with 1×PBS buffer followed by incubation in different concentrations of FITC labeled monomeric or heptameric targeting ligand for 30 min at room temperature. The coverslips were washed three times with 1×PBS. The resulting samples were visualized by using Zeiss LSM 510 confocal microscope at the UNC microscopy core facility.

### Flow cytometry

Cell binding of the monomeric and heptameric ligands was evaluated by using flow cytometry. Approximately 2×10^5^ cells per sample were washed with PBS. Cells were incubated with FITC-labeled monomeric or heptameric ligand for 30 min at room temperature, followed by washing with PBS twice. The samples were analyzed by flow cytometer (BD FACS Canto flow cytometry) and the data were analyzed by Flow Jo system (Tree star, Inc. Ashland, OR).

### BIAcore analysis

The BIAcore 2000 (BIAcore AB, Uppsala, Sweden) was used for surface plasmon resonance analysis. 1 µg of purified extracellular domain of recombinant human EGFR ECD-Fc, HER2 ECD-Fc, or PSMA (R&D System, Minneapolis, MN), was diluted in a buffer containing 10 mM sodium acetate pH 5.0 and immobilized on CM5 sensor chip (GE healthcare, Piscataway, NJ) by amine coupling according to the manufacturer's instruction (about 2,500 resonance units). Various concentrations of monomeric or heptameric ligands were injected onto the flow cell in an HBS-P buffer (10 mM HEPES pH 7.4, 150 mM NaCl, and 0.005% surfactant P20) at a flow rate of 20 µl/min. The dissociation equilibrium constant (K_D_), the association rate (K_a_), the dissociation rate (K_d_) were calculated using the BIAevaluation software (BIAcore) by fitting the data on a one to one Langmuir binding model.

### Circular dichroism spectroscopy

Highly purified monomeric or heptameric proteins were prepared in 10 mM phosphate buffer (pH 7.5) and used for circular dichroism (CD) scanning with an AVIV model 202-01 spectropolarimeter at the UNC macromolecular interaction facility. Spectra were recorded from 190 nm to 260 nm at 0.2 nm intervals, a scan speed of 20 nm/min, a bandwidth of 2 nm, and an integration time of 1 s. To determine thermal stability, spectra were recorded by gradually increasing the temperature from 25°C to 94°C at 220 nm.

### Resistance to protease-mediated degradation

The protease digestion was performed in HBS buffer (10 mM HEPES pH 7.4 and 150 mM NaCl) at 25°C, 37°C, 42°C, and 60°C, respectively, for 20 min. About 5 µg of each protein was incubated with 100 ng of thermolysin. After incubation, the resulting reaction mixtures were separated by SDS-PAGE to monitor the extent of protein degradation.

### Co-localization studies

A431 and SK-OV3 cells were seeded onto the coverslips and grown for 16 h at 37°C. FITC-labeled 10 nM or 100 nM of heptameric Z^EGFR^ and Z^HER2^ were incubated for 2 h at 37°C with A431 and SK-OV3 cells grown on coverslips, respectively. After washing away unbound targeting ligands, the cells were fixed with 2% paraformaldehyde in PBS for 15 min at room temperature. Cells were then washed three times with 1×PBS. For immunostaining, blocking solution (PBS with 5% BSA and 0.3% Triton X-100) was added and incubated for 1 h at 4°C. The cells were then incubated with the anti-EEA1 rabbit monoclonal antibody (1:200) (Cell Signaling Technology, Danvers, MA) overnight at 4°C. After incubation with secondary antibody (Alexa Fluor 555 conjugated anti-Rabbit IgG (Cell Signaling Technology, Danvers, MA) for 1 h at 4°C, the corresponding cells were rinsed three times with 1×PBS followed by the addition of an antifade reagent. Cells were examined by using Zeiss LSM 510 confocal microscope at the UNC microscopy core facility.

### Cell proliferation MTS assay

CellTiter96 Aqueous Non-Radioactive Cell Proliferation Assay kit from Promega (Madision, WI) was used for the MTS assay. Approximately 1×10^4^ cells were seeded in each well of a 96-well plate and grown for 16 h at 37°C. Each heptameric molecule was incubated with the cells for 24 h. 4 µM Cis-platinum (II) diamine dichloride (Sigma-Aldrich Chemical Co, St Louis, MO) was used as a positive control. Approximately 20 µl of MTS/PMS solution was added into each well followed by incubation for 4 h at 37°C. The absorbance at 490 nm was recorded using an ELISA plate reader.

## Results

### General design of heptameric targeting ligands

In the previous self-association approaches for the generation of trimeric and pentameric complexes, additional cysteine (Cys) residues were introduced to stabilize the oligomeric structure through the formation of inter-molecular disulfide bonds [10], [11], [14], [17]. However, the formation of undesired disulfide bonds is common, resulting in mis-folding, aggregation, and loss of target-binding functions [1]. To circumvent these problems, we decided to use the 70-amino acid AF-Sm1 heptamerization domain from hyperthermophilic *Archaeoglobus fulgidus*, which is highly stable and can efficiently self-assemble into a parallel heptameric complex without relying on any disulfide bond(Figure 1).

The general strategy we used to develop the heptameric targeting ligands is to fuse a small target-binding protein domain through a hinge linker with the AF-Sm1 heptamerization domain (Figure 1A). To investigate whether functional heptameric targeting ligands can be readily generated using this strategy, we used an EGFR- or HER2-binding affibody, Z^EGFR^ or Z^HER2^, that does not contain any Cys residue, as reported in the literature, to facilitate the self-assembly process [18], [19]. The affibody is composed of 58-amino acid derived from the immunoglobulin binding Z-domain of *staphylococcal* protein A [18], a small (∼7 kDa) protein domain with a three-helix bundle structure (Figure 1B). It has been extensively reported that the affibody could be selected with high affinity to any given target from a library with high diversity [18], [19]. To compare the monomeric and the corresponding heptameric forms, the monomeric targeting ligands were constructed by deleting the heptamerization domain (Figure 1A). In addition, a His-tag was introduced at the C-terminus of both monomeric and heptameric ligands to facilitate protein purification.

### Expression, purification and characterization of monomeric and heptameric targeting ligands

The cDNAs that encode the monomeric and heptameric targeting ligands were cloned into a pET28b expression vector that contains a C-terminal 6×His-tag to facilitate protein purification by Co^2+^-nitrilotriacetic acid (NTA) column. The expression level of the targeting ligands was high with a yield of approximately 20 mg/L and 10 mg/L for the monomeric and heptameric ligands as soluble proteins, respectively. Co^2+^-NTA purified monomeric and heptameric ligands were analyzed by SDS-PAGE (Figure 2). The predicted molecular weight of the monomeric targeting ligands without the heptamerization domain is about 10.2 kDa for Z^EGFR^ and 10.5 kDa for Z^HER2^, respectively. In the case of heptameric ligands, the monomeric form with the AF-Sm1 domain has a molecular weight of 18 kDa, whereas its corresponding heptameric form has a predicted molecular weight of 126 kDa. As shown in Figure 2, both monomeric Z^EGFR^ and Z^HER2^ could be purified to near homogeneity. When a heptamerization domain was introduced in the construct, the vast majority of the expressed protein was present in a multimeric form with molecular weight of approximately 130 kDa, even though a small portion of the monomeric form (18 kDa) was also detected (Figure 2). Because the molecular weight of the multimeric form (130 kDa) is very close to that of the putative heptameric form (126 kDa), it strongly suggests that this multimeric form is the putative heptamer. It appears that the formation of the heptameric form was efficient without applying any special folding procedures, as shown by SDS-PAGE (Figure 2). The heptameric form is highly stable since it can resist the strong denaturing conditions of SDS present in the loading buffer as well as in the polyacrylamide gel. This result clearly indicates that the self-assembly to a heptameric form is robust and highly efficient. The presence of a small amount of the monomeric form on SDS-PAGE raises question whether the monomeric form co-exists with the heptameric form before SDS-PAGE analysis or it is generated by disassembling the heptameric form back to the monomeric form when denaturing conditions were applied during SDS-PAGE. To address this question, we further examined the purified heptameric ligands by using native gel electrophoresis. As shown in Figure 3, both purified heptameric Z^EGFR^ and Z^HER2^ targeting ligands were present as a single band under non-denaturing conditions with much lower mobility compared with monomeric ligands, whereas the corresponding 18 kDa monomeric form was not detected. Taken together, the self-assembled multimeric targeting ligands exist predominately as a heptameric form under native conditions.

Although the molecular weight of the heptameric form on SDS-PAGE is around 130 kDa, it is of great interest to measure the exact molecular weight of the putative heptamer. To further confirm the heptameric state, we used analytical ultracentrifugation to determine the molecular weights of both heptameric ligands. As shown in Figure 4, the two putative heptameric targeting ligands have a molecular weight of 131±3 kDa for heptameric Z^EGFR^ and 130±2 kDa for heptameric Z^HER2^, respectively. These values are consistent with those shown from SDS-PAGE gels and also match the theoretical molecular weights (∼126 kDa) of the heptameric form. Altogether, we concluded that the multimerization domain containing targeting ligand can self-assemble into a heptameric form very efficiently under native conditions while the presence of the monomeric form is minimal.

An ideal affinity molecule should have exceptional stability to be readily used in various *in vitro* and *in vivo* applications. To determine the thermal stability of these targeting ligands, we performed circular dichroism (CD) analysis using highly purified monomeric and heptameric proteins. Thermal denaturation was monitored at 220 nm. As shown in Figures 5A and 5B, the T_m_ value of each protein is approximately 65°C for all ligands, whereas the heptameric complex itself is highly resistant to heat-induced denaturation (Figure 5C). These results indicate that heptameric Z^EGFR^ and Z^HER2^ ligands are as stable as monomeric ligands. Degradation of protein-based affinity molecules by various physiological proteases is another barrier that must be overcome for their *in vivo* applications. To examine the resistance of these targeting ligands to proteases, we performed a protease-mediated digestion assay by subjecting the monomeric or heptameric targeting ligands to a thermostable metallopeptidase thermolysin according to the procedure we have reported [20]. All of the heptameric forms were resistant to thermolysin digestion even when the temperature was as high as 60°C, whereas monomeric forms are more susceptible to protease at 60°C(Figures 6A and 6B). This result demonstrates that such heptameric targeting ligands are stable under harsh conditions, implying that they have a higher potential of being resistant to degradation *in vivo* and used as targeting ligands for *in vivo* applications.

### The determination of target binding strength

To investigate whether purified heptameric complex maintained the target binding features towards the target of interest, surface plasmon resonance (SPR) was employed to examine the binding strength and specificity of the monomeric and heptameric ligands. Here, we immobilized the extracellular domains of EGFR or HER2 on the surface of CM5 biosensor chip followed by injection of purified monomeric or heptameric ligands. No binding was detected for any of the targeting ligands when an irrelevant protein target, such as PSMA (Prostate Specific Membrane Antigen), was used as a negative control (data not shown). As expected, it was evident that Z^EGFR^ targeting ligands did not bind to HER2 receptor, and neither Z^HER2^ targeting ligands showed any detectable binding against EGFR (data not shown). The binding constant K_D_ of the monomeric Z^EGFR^ ligand (2.6±0.3 nM) by fitting data on one to one Langmuir binding model was similar to that of affibody (5 nM) reported by Stahl and co-workers (Table 2 and figure 7A) [19]. The heptameric Z^EGFR^ ligand has greatly enhanced EGFR-binding strength at K_D_ of 29±20 pM, which is approximately 100 fold higher than that of the monomeric form (Table 2). In the case of the heptameric Z^HER2^ ligand, about 1000 fold increased HER2-binding strength (K_D_ of 2 ±0.5 pM) was achieved compared to that of the monomeric Z^HER2^ ligand (K_D_ of 1.7±0.7 nM) (Table 2 and Figure 7B). These results clearly indicate that the target binding strengths of heptameric ligands have significantly increased as a result of the multivalency effect.

### Binding of monomeric and heptameric targeting ligands with cell surface biomarkers

The multivalent binding effect of heptameric ligands depends on many factors, particularly the density of target receptors on the cell surface. The density of each target receptor on CM5 chip is quite different from that on the surface of live cells. Although we used purified proteins originated from mammalian cells to circumvent post-translational modification issues, the SPR experimental conditions cannot mimic the *in vivo* cellular conditions and therefore this warranted further investigation of the heptameric ligands through cell surface binding assays. To investigate the target-binding properties of each ligand using cell lines that overexpress native EGFR or HER2, we labeled each targeting ligand with FITC to visualize its binding with cells. For monomeric and heptameric Z^EGFR^ ligands, we used A431 cells that overexpress EGFR, while EGFR-negative Jurkat cells were used as a negative control. The binding signal can be easily detected on EGFR-expressing A431 cells when both monomeric and heptameric Z^EGFR^ ligands were used (Figure 8A). In contrast, minimal fluorescent signal was observed on Jurkat cells when either monomeric or heptameric Z^EGFR^ ligand was used, even when the concentration of the targeting ligand was as high as 100 nM (Figure 8C). The cell binding strength of heptameric Z^EGFR^ ligand is much stronger than that of the monomeric ligand. As shown in Figure 8A, 10 nM of monomeric Z^EGFR^ ligand was required to achieve the same cell binding signal compared to 0.1 nM of heptameric Z^EGFR^ ligand. We also compared the cell-binding properties of the monomeric and heptameric Z^HER2^ targeting ligands by using SK-OV3 cells that highly express HER2, while MCF7 cells with a much lower HER2 expression level were used as a negative control. As shown in Figure 8B, as low as 0.1 nM heptameric Z^HER2^ ligand bound to HER2-positive SK-OV3 cells, whereas more than 10 nM monomeric Z^HER2^ ligand was required to achieve comparable results (Figure 8B). However, no detectable binding to MCF cells was observed even when 100 nM of monomeric or heptameric ligand was used (Figure 8C). These results were consistent with the SPR data that both monomeric and heptameric Z^HER2^ ligands bound HER2 receptor specifically. Taken together, these observations indicate that heptameric targeting ligands can specifically bind to their cell surface biomarkers and their binding strengths are at least 100 fold higher than the monomeric ligand. Additionally, we have conducted cell binding analysis by using flow cytometry. The 100 nM FITC-labeled monomeric and heptameric Z^EGFR^ ligands were incubated with A431 and Jurkat cells, respectively. No binding was detected on EGFR negative Jurkat cells (Figure 9A). However, strong binding signal was detected on EGFR positive A431 cells by using both monomeric and heptameric ligand (Figure 9A). Heptameric Z^EGFR^ ligand bound to A431 cells about 10 fold stronger than monomeric Z^EGFR^ ligand (Figure 9A). Similarly, the 100 nM FITC- labeled monomeric and heptameric Z^HER2^ ligands were incubated with SK-OV3 and MCF7 cells, respectively. Both monomeric and heptameric ligands bound to SK-OV3 strongly and maintained target receptor binding specificity even though the heptameric Z^HER2^ ligand bound to MCF7 weakly (Figure 9B). It was also confirmed that heptameric Z^HER2^ bound to SK-OV3 cells stronger than monomeric Z^HER2^ (Figure 9B). According to flow cytometry analysis, both heptameric ligands bound stronger to their target receptor compared to the corresponding monomeric ligands. It is possible the weak binding of heptameric Z^HER2^ ligand to MCF7 cells, which express low level of HER 2, is due to the avidity effect of the heptameric ligand. Collectively, target binding specificity of each heptameric ligand was well maintained along with strong binding affinity.

### Heptameric targeting ligands are internalized and present in the endosome

One of exciting applications of targeting ligands is the delivery of imaging or therapeutic agents into specific cell types. Therefore, it is important to study whether targeting ligands can be internalized and to further investigate the sub-cellular localization of the internalized molecules. It is well known that the binding of EGF to EGFR promotes the internalization of the receptor through the endocytic pathway, and the internalized EGFR is strongly associated with an early endosome marker Early Endosome Antigen 1(EEA1) that is enriched in endosomes [21]. The monomeric and heptameric Z^EGFR^ ligands used in this study contain the same affibody (Z1907) as reported in the literature [22]. Recently, it was reported by Frejd and co-workers that monomeric and dimeric EGFR-binding affibody (Z1907) were internalized as efficiently as EGFR monoclonal antibody cetuximab when they were incubated with A431 cells [22], [23].

To investigate the internalization and subsequent sub-cellular location of heptameric Z^EGFR^ ligand, the targeting ligand was incubated with A431 cells at 37°C for 2 h to promote its internalization. As illustrated in Figure 10A, bright punctuated dots can be observed using confocal microscopy, demonstrating that FITC labeled heptameric Z^EGFR^ (green fluorescence) was internalized and co-localized (white arrows) with the early endosome marker EEA1 (red fluorescence). However, some of the heptameric ligand signal (green) did not overlap with EEA1 (red). It is possible that some of heptameric ligands might escape from endosome or localize within late endosome that cannot be detected through EEA1. Similarly, the sub-cellular localization of heptameric Z^HER2^ ligand was also investigated by incubating FITC labeled heptameric Z^HER2^ ligands with SK-OV3 cells. It appears that heptameric Z^HER2^ ligand also co-localized with EEA1 in endosomes (Figure 10B), but more heptameric Z^HER2^ ligands are present on cell surface when compared to the heptameric Z^EGFR^ ligand. Our results are consistent with previous finding that HER2 endocytosis and HER2 mediated uptake of affibody were slower than that of EGFR [24], [25]. These findings clearly demonstrated that both heptameric targeting ligands were internalized and co-localized with EEA1in endosome.

### Cellular toxicity of the heptameric targeting ligands

The internalization property of the heptameric ligands may be used for the intracellular delivery of a variety of agents such as anti-cancer drugs and imaging agents. However, an ideal targeting ligand must have minimal cellular toxicity. To examine the degree of cellular toxicity of the heptameric ligands, their effect on cell proliferation was assessed by the MTS assay. Various concentrations ranging from 250 nM to 4 µM of an appropriate heptameric ligand was incubated with EGFR-positive A431 or HER2-positive SK-OV3 cells. The anti-cancer drug cisplatin that interferes with cancer cell growth by inhibiting DNA metabolism through its binding to DNA was used as a positive control [26]. Figure 11 illustrates that heptameric ligands showed very little cell growth inhibition (at most 4%), while cisplatin at 4 µM inhibited cell proliferation to 50%. These results indicate that the heptameric targeting ligands by themselves are not toxic to cells at least under the conditions we have investigated (at concentrations up to 4 µM).

## Discussion

The role of multivalency in enhancing affinity of a ligand towards its target has been well studied [1]. The ideal targeting ligand with desired multivalency should be composed of multiple target-binding moieties displayed in parallel surrounding a multimeric core with unusually high stability. However, the generation of novel multivalent targeting ligands with desired properties, such as high stability and significantly improved functional affinity, is a difficult task. Here, we report the first successful generation of heptameric targeting ligands, against EGFR and HER2 receptor, using the heptamerization domain from the Archaeal RNA binding protein (Figures 1A and 1B). On the basis of our findings, it appears that the AF-Sm1 domain has several unique advantages to serve as an ideal scaffold for heptamerization of targeting ligands. These features include its cysteine-free amino acid sequence, spontaneous and highly efficient self-assembly process, exceptionally high stability against heat and protease-induced degradation, economical and high expression level in *E. coli*, and non-existing cellular toxicity.

In addition to a robust heptameric core, it is necessary to have an affinity moiety and a flexible linker to make a functional heptameric targeting ligand possible. We decided to use previously well-characterized, widely used EGFR- and HER2-binding affibodies (∼7 kDa) as the affinity molecules [18], [19]. These affibodies were previously selected by phage display that bound the extracellular domain of EGFR or HER2 with sub-nanomolar affinities [18], [19]. Unlike display of short peptides with just a few amino acids, display of seven protein domains at the top of a heptameric core without affecting the right geometry is challenging. The AF-Sm1 domain-based heptameric core is very compact and rigid, with the outer and inner diameters of 65 Å and 13 Å, respectively [16]. To facilitate the parallel display of seven AF-Sm1 domains, we introduced a flexible hinge linker between the target binding moiety and the heptamerization domain, which presumably provides greater inter-unit spacing so that each monomeric Z^EGFR^ or Z^HER2^ can be properly folded without disrupting the heptameric complex. Since multiple affinity units are present to the targets on cell membrane at a very close distance, the free energy of target binding with the heptameric ligand should be much higher than that with the monomeric ligand. Indeed, significantly improved target-binding strength of the heptameric ligands was observed when compared to the respective monomeric ligands (Figure 7 and Table 2).

Both heptameric Z^EGFR^ and Z^HER2^ ligands were highly stable as demonstrated by its efficient formation even under harsh denaturing conditions, such as SDS- or heat-induced denaturation (Figures 2, 5 and 6). No detectable monomeric ligand was observed under native conditions (Figure 3), suggesting that the small amount of monomeric form observed in the SDS-PAGE gels is likely due to the dissociation of a small proportion of the heptamer under denaturing conditions (Figure 2). Moreover, the high stability of heptameric ligands was demonstrated by CD analysis and protease-mediate degradation (Figure 5 and 6). Both heptameric ligands were as stable as their monomeric counterparts as the temperature was raised to 94^°^C during CD analysis (Figures 5A and 5B). The minimal degradation by thermolysin, a highly active protease at high temperature, provided further evidence that the heptameric ligands have exceptional high stability (Figures 6A and 6B). The Archaeal derived heptamerization domain has been evolutionarily selected to withstand extreme environmental conditions such as high temperature and low pH [15]. The exceptional high stability of AF-Sm1 can be well-explained by examining its X-ray crystal structure, in which the doughnut shaped heptameric ring is extensively stabilized by the inter-subunit β sheet hydrogen bonding and the combination of hydrophobic and electrostatic interactions at the interface of each monomeric subunit [16]. Each heptameric ligand maintains its target-binding specificity without cross reactivity (Figures 7A and 7B; and Table 2). The *in vitro* target-binding strength of the heptameric ligands was significantly enhanced (up to 1000 fold) compared to the monomeric ligands as shown by SPR studies (Figure 7 and Table 2). This is presumably due to the greatly reduced dissociation rate, which is slowed down 280 and 4,000 times in heptameric Z^EGFR^ and Z^HER2^, respectively (Table 2). However, cell binding analysis indicated that heptameric ligands showed only 100 fold increase in binding with EGFR- or HER2-positive cells (Figure 8). The discrepancy of binding strength between *in vitro* and *in vivo* analyses is typical as observed in many other targeting ligands. First, the density and the accessibility of a receptor on the cell surface are quite different from that on the CM5 chip. The presence of various other receptors and proteins on the cell membrane should result in limited accessibility of the cell surface receptor by its ligand. Another possibility for the marginal difference between monomeric and heptameric ligands in cellular studies is that the affinity of the monomers we used is already in the range of low nanomolars, making it difficult to further increase through multivalency [27]. Furthermore, the covalent labeling of targeting ligands with FITC for *in vivo* cellular studies may lead to the modification of some residues that are critical for targeting binding, consistent with the report by Lyakhovin the studies of interaction between EGFR-binding affibody and its receptor [28].

The receptor-bound heptameric ligands were efficiently internalized and further co-localized with the early endosome marker EEA1. This finding indicates that the heptameric system can be utilized as a carrier for intracellular delivery of various anticancer agents. Indeed, we have demonstrated recently that nickel nanoparticles (Ni-NP) conjugated with heptameric Z^EGFR^ ligand can be used for efficient targeting EGFR-positive A431 cells, with more than 9-fold increase of cellular uptake compared to untargeted nanoparticles [29]. Furthermore, the heptameric Z^EGFR^ ligand could facilitate *in vivo* accumulation of Ni-NPs in nude mice bearing A431 cells [29]. These results support our hypothesis that the heptameric targeting system described here has the great potential to be widely used for various *in vivo* applications.

Compared with our previous pentameric targeting ligand, it appears that the heptameric ligands do not increase the target-binding strength to more than 10^4^ [20]. However, it is difficult to directly compare these systems since different scaffolds of the targeting moieties were used. Nevertheless, the heptameric system has several unique advantages. First, the heptameric targeting ligand exists predominantly as a heptamer without any detectable intermediate forms. This is very different from the pentameric ligand that is present as a mixture of tri-, tetra-, and pentameric forms that complicate the purification process [20]. Second, the spontaneous and highly efficient self-assembly of the heptameric ligand is totally independent on the disulfide bond(s), but relies on the extensive inter-subunit hydrogen bonding, hydrophobic and electrostatic interactions. In contrast, the pentameric complexes rely on the critical inter-subunit disulfide bonds to maintain its multimeric structure. Third, the cysteine-free nature of the heptameric ligand can greatly facilitate its site-specific conjugation with other biomolecules, such as anticancer agents or nanoparticles, by introduction of the only cysteine at the N- or C-terminus. The facile and economic generation of these high-avidity affinity molecules makes them a valuable complement to the conventional antibody-based targeting ligands for both *in vitro* and *in vivo* applications. One additional advantage of the heptameric system is the spontaneous increase of the molecular weight from 18 kDa in monomer to greater than 130 kDa in heptamer, which could presumably extend the *in vivo* half-life of these ligands by reducing kidney clearance.

In summary, our results demonstrate that heptameric targeting ligands with high stability, enhanced avidity, and non-toxicity can be easily generated through a facile and highly efficient self-assembly process. Although the heptameric targeting ligands we demonstrated here are for binding with EGFR or HER2 receptors, the same approach should be generally applied to the rapid generation of high-avidity affinity molecules based on other target-binding moieties such as short homing peptides, single domain antibody mimics, and natural antibody fragments. Our current and future work will explore the application of these heptameric molecules for the targeted delivery of anticancer agents. It is worth mentioning that the AF-Sm1 domain is a robust RNA-binding complex [15], making it possible to use the heptameric ligands described here for the targeted delivery of nucleic acid drugs simply by add-and-mix strategy.
